# Development of a composite regional vulnerability index and its relationship with the impacts of the COVID-19 pandemic

**DOI:** 10.1007/s43762-023-00078-x

**Published:** 2023-01-16

**Authors:** Mengqiu Cao, Qing Yao, Bingsheng Chen, Yantao Ling, Yuping Hu, Guangxi Xu

**Affiliations:** 1 University of Westminster, London, UK; 2 Beijing Normal University/ Imperial College London, Beijing, China; 3 Imperial College London, London, UK; 4 Chongqing University of Technology, Chongqing, China; 5 The People’s Hospital of Shapingba District, Chongqing, China; 6 The Affiliated Hospital of Xuzhou Medical University, Xuzhou, China

**Keywords:** Vulnerability index, Travel vulnerability, Housing vulnerability, Social deprivation, Greater London, COVID-19

## Abstract

The interactions between vulnerability and human activities have largely been regarded in terms of the level of risk they pose, both internally and externally, for certain groups of disadvantaged individuals and regions/areas. However, to date, very few studies have attempted to develop a comprehensive composite regional vulnerability index, in relation to travel, housing, and social deprivation, which can be used to measure vulnerability at an aggregated level in the social sciences. Therefore, this research aims to develop a composite regional vulnerability index with which to examine the combined issues of travel, housing and socio-economic vulnerability (THASV index). It also explores the index’s relationship with the impacts of the COVID-19 pandemic, reflecting both social and spatial inequality, using Greater London as a case study, with data analysed at the level of Middle Layer Super Output Areas (MSOAs). The findings show that most of the areas with high levels of composite vulnerability are distributed in Outer London, particularly in suburban areas. In addition, it is also found that there is a spatial correlation between the THASV index and the risk of COVID-19 deaths, which further exacerbates the potential implications of social deprivation and spatial inequality. Moreover, the results of the multiscale geographically weighted regression (MGWR) show that the travel and socio-economic indicators in a neighbouring district and the related vulnerability indices are strongly associated with the risk of dying from COVID-19. In terms of policy implications, the findings can be used to inform sustainable city planning and urban development strategies designed to resolve urban socio-spatial inequalities and the potential related impacts of COVID-19, as well as guiding future policy evaluation of urban structural patterns in relation to vulnerable areas.

## Introduction

On the 2030 agenda for sustainable development, the United Nations proposed 17 sustainable development goals (SDGs) for both developing and developed countries, which need to be acted on urgently (UN, 2018). Most countries have recognised the importance of these issues and have consequently tried to mitigate deprivation, inequality and poverty. However, it is imperative that as much as possible is done quickly, using various strategies to boost economic growth, reduce the gap between rich and poor, and improve education and public health while also protecting the environment. Nonetheless, it can be argued that it is still very difficult to persuade every country to actively engage in achieving the SDGs, particularly during the current COVID-19 global pandemic. In order to address some of the SDGs, such as good health and well-being (SDG3); reduced inequalities (SDG10); sustainable cities and communities (SDG11); and peace, justice and strong institutions (SDG16) (UN, 2018), we developed a composite THASV index that can be used to measure vulnerability with regard to both social and spatial inequality issues in a city. Generally, vulnerability is defined as the sensitivity of an individual or community to suffering damage from adverse events. Previous studies have attempted to evaluate individual and/or community vulnerability to one type or all types of disturbances, and vulnerability has been defined in different ways in different contexts. In this study, given that the aim of the research is to primarily focus on the identification of vulnerable areas at an aggregated level within a city, vulnerability is defined as meaning that people are more likely to experience the combined issues of car dependence, housing affordability and social deprivation if they live in areas of a city that have been identified as potentially vulnerable (see, and developed from, Cao & Hickman, 2018; Leung et al., 2018; Dodson, Sipe and Li, 2015).

Most studies have primarily investigated the impacts of transport disadvantage (Carroll et al., 2021; Oviedo & Sabogal, 2020), housing affordability (Edwards, 2016; Gallent, 2016), and social inequality and poverty (Deutsch et al., 2020; Lai & Taylor-Robinson, 2018; Moro et al., 2021), respectively, rather than using a simple composite vulnerability index to assess the overall vulnerability of individuals at an aggregated level.

For example, Cao and Hickman (2018) argued that, when examining travel vulnerability, researchers should also take housing affordability into account, as the high cost of housing means that some residents may be forced to move further away from the centre and live in outer suburban areas of Greater London (or even beyond the M25) where housing is somewhat cheaper. Therefore, they developed a means of measuring high levels of car dependence and housing affordability combined, in view of the fact that petrol/diesel prices were likely to become more volatile and housing prices would continue to rise (Cao & Hickman, 2018). It can also be argued that housing affordability is not perceived as a ‘transport’ issue, or as related to travel vulnerability, even though it has an impact on travel behaviours, which in turn has a significant influence on the overall vulnerability of individuals. Similar work has also been carried out to investigate and develop simple ways of constructing combined vulnerability indices, for example, to assess rises in the price of petrol and urban transport oil vulnerability (Dodson & Sipe, 2007; Leung et al., 2018; Lovelace & Philips, 2014); vulnerability in regard to mortgage, petroleum, and inflation risks and expenditure (Dodson & Sipe, 2008; Dodson, Sipe and Li, 2015); transport poverty and the resulting adverse social consequences (Lucas et al., 2016); and the processes of gentrification and displacement (Chapple & Zuk, 2016; Zhang et al., 2020).

However, to the best of our knowledge, there have been no attempts to develop a simple approach that can be used to assess the combined issues of travel, housing and socio-economic vulnerability (THASV index), and which reflects socio-spatial inequalities at an aggregated level. There is an index of multiple deprivation which covers seven domains of deprivation, namely: the living environment, barriers to housing and services, crime, health, education, employment, and income (MHCLG, 2019), but it only applies to the UK and cannot be easily replicated and applied to other countries or international cities. Therefore, it is worth constructing a combined vulnerability index, which has the ability to reflect socio-spatial inequalities at an aggregated level. In this research, we use Greater London as a case study, with data analysed at the level of Middle Layer Super Output Areas (MSOAs).

The following three research questions were developed to achieve the aforementioned aim:

- Can a comprehensive vulnerability index be developed to assess the combined issues of car dependence, housing affordability and social deprivation in a city?
- What are the potential spatial relationships between the THASV index and COVID-19 deaths that can be measured at a geographical level?
- What are the key factors that result in a higher risk of COVID-19 infection/deaths?

The remainder of the study is organised as follows: Section 2 reviews the existing literature on vulnerability. Section 3 describes the data and the methods used. Section 4 presents the findings and a discussion derived from the statistical and spatial analyses. The last section summarises the study and highlights both the key theoretical and methodological contributions, as well as suggesting policy implications.

## Literature review

The concept of vulnerability can be traced back to the seventeenth century, and can be divided into two broad approaches: the former perceives vulnerability in terms of humanity; while the latter understands it from a universal perspective (Mackenzie et al., 2014). According to this concept of vulnerability, it is argued that the effects of human daily activities should be treated as equally important to those of natural disasters in relation to vulnerability (Blaikie et al., 2004; Fekete, 2019). In general, human vulnerability primarily focuses on the relationships between human activities and the natural environment (Kabbani & Olds, 2021; Wu et al., 2021); virtual embodiment (e.g. health, illness or death) (Pinato et al., 2021; Tiwari et al., 2021); and socio-demographic/socio-spatial and political factors (Karácsonyi et al., 2021; Song et al., 2021; Zhang & Cao, 2020).

With regards to human vulnerability and the external natural environment, Nguyen and Liou (2019) proposed an assessment framework comprising 16 indicators classified into 6 levels that can be used to quantify and map global eco-environmental vulnerability (GEV), in terms of both man-made and natural disturbances. They found that Ethiopia, India and China have relatively high eco-environmental levels of vulnerability (Nguyen & Liou, 2019). In addition, Polsky et al. (2007) developed a vulnerability scoping diagram (VSD) model which they divided into three main dimensions, namely adaptability, sensitivity and exposure. It offers a basic vulnerability assessment framework with which to draw comparisons between various independent and multiple dissimilar measures. Furthermore, Han et al. (2020) developed a human-environment system vulnerability index, based on the earlier work of Polsky et al. (2007), comprising 33 indicators and 14 factor layers, which they applied to a case study of Ningxia. The results showed that the overall vulnerability level of the human-environment system was high across different geographical areas, and was exacerbated by human-induced high-intensity activities, natural disasters and a fragile ecological environment.

In terms of virtual embodiment, Sasidharan et al. (2020) developed a vulnerability-based approach and applied a linear regression model to examine the relationship between air pollution (PM_2.5_ and NO_2_), and the reported risk of COVID-19 fatalities for various London Boroughs. They demonstrated a strong association between higher levels of air pollution and a higher risk of COVID-19 deaths on a regional scale. Meanwhile, Tiwari et al. (2021) employed a machine learning approach to develop a COVID-19 vulnerability index (C19VI) to measure the heterogeneity of county level vulnerabilities in the U.S., which could be used as a risk evaluation tool to inform and implement policies designed to control the spread of the COVID-19 pandemic. Baggio et al. (2021) carried out a cross-sectional study of 59,695 cases of COVID-19 in the state of Alagoas in Brazil, using different types of datasets, such as clinical-epidemiological variables, COVID-19 case fatality, mortality and incidence rates, and the social vulnerability index and municipal human development index, to measure spatial risk and social vulnerability. Being male, an older adult, and the presence of comorbidities were found to be potential predictors of death in patients with COVID-19.

With regard to socio-economic and socio-spatial vulnerabilities, only a few studies within the social sciences have proposed a simple and innovative approach for assessing these, using comprehensive indicators. For instance, a case study of social vulnerability and flooding disasters in Nanjing - a city in a developing country - was conducted by Chen et al. (2021), who found that affordable housing communities located further away from the city centre were more likely to have a relatively lower level of flood vulnerability. Furthermore, Tanir et al. (2021) used an exposure index which they combined with the socio-economic vulnerability index (SOVI) to identify groups who were vulnerable to compound flood events in the Washington DC metropolitan area. However, it can be argued that flooding disasters rarely occur in cities in developed countries, such as London. Therefore, it may be very useful to apply or replicate a vulnerability indicator relating to urban flooding, if one is attempting to address socio-economic issues. In addition, vulnerability and social deprivation relating to travel and housing affordability have a substantial impact on people’s everyday life; however, limited research has addressed the aforementioned issues by developing a composite index with which to assess the risk of vulnerability for individuals at an aggregated level. For example, Leung et al. (2018) constructed a composite urban transport oil vulnerability index, comprising the components of exposure, sensitivity and adaptive capacity, to evaluate and map car usage-related oil vulnerability in Hong Kong and Brisbane. Mattioli et al. (2019) proposed a composite socio-spatial vulnerability index which combined data on vehicle registration, vehicle inspection, accessibility and income for England in 2011, and found that vulnerability to increases in motor fuel prices was higher in the peri-urban North of England. Furthermore, an index for measuring vulnerability to rises in petrol expenses (VIPER) and vulnerability assessment indices for mortgage, petrol and inflation risks and expenditure (VAMPIRE) were developed by Dodson and Sipe (2007), Dodson and Sipe (2008) and Dodson et al. (2015), respectively, to assess vulnerabilities to oil and mortgage price increases in Australian cities. Based on the approach applied by Dodson et al. (2015), Cao and Hickman (2018) developed a new composite car dependence and housing affordability (CDHA) index to measure vulnerability to car dependence and housing affordability; however, it did not include indicators relating to public transport and social deprivation, and nor did it examine the relationship between vulnerability and the current COVID-19 pandemic.

Therefore, based on the aforementioned existing literature, it can be seen that the interactions between vulnerability and human activities have generally been examined in terms of the level of risk they pose, both internally, i.e., risks relating to man-made or human activity, particularly for disadvantaged groups at a disaggregated level; and externally, i.e., risks relating to natural disasters such as earthquakes and flooding in specific regions at an aggregated level. However, to date, few if any studies have attempted to develop a simple and comprehensive composite regional vulnerability index, which can be used to measure, for instance, socio-spatial vulnerability,^1^ and which specifically combines issues relating to travel, housing and socio-economic and socio-spatial aspects. In addition, most studies have focused on assessing the relationship between natural disasters/hazards and social vulnerability (Cutter et al., 2009, 2012; Derakhshan et al., 2020; Zarghami & Dumrak, 2021), but very few have used a variety of different indicators to measure socio-spatial vulnerability, particularly in the UK and European cities. Therefore, this study aims to fill the research gaps by developing a composite vulnerability index, specifically designed to examine the combined issues of travel, housing and socio-economic vulnerability (THASV index), as well as exploring its relationship with the current impacts of the COVID-19 pandemic (Arsalan et al., 2020; Zhang & Cao, 2020), reflecting both social and spatial inequalities, using Greater London as a case study, with data analysed at the level of Middle Layer Super Output Areas (MSOAs).

## Methodology and data

### Methods

Equation (1) represents the THASV index, which allows a composite analysis of the combined issues of travel, housing and social vulnerability to be calculated for each MSOA area. In addition, in order to develop the mapping, construct the composite THASV index and carry out a spatial analysis and comparison based on the same scale, we converted the original values into an index ranging from point 1 to point 10 (classification method via Python: ‘Quantile-based discretisation function’ in a geographical sense). For example, point 1 indicates minimal vulnerability shaded in light red, and point 10 means extremely high vulnerability shaded in dark red on the map (see Fig. 2i). In terms of variable weighting, there is relatively limited discussion in the existing literature about which variables should be allocated a higher weighting. It may also be necessary to assign the weightings differently depending on the application and objective(s). The discussion is inconclusive with regard to whether particular variables should be given priority, and prioritisation is, therefore likely to vary by individual preference. Thus, in this study, we broadly followed the approach previously used by Cao and Hickman (2018), Dodson and Sipe (2007), Dodson and Sipe (2008), Dodson et al. (2015), and Leung et al. (2018), and experimented with possible weights in combination with the variables for the data-driven optimisation. Based on the results, we decided to assign an equal 50% weighting to both travel vulnerability and housing and social vulnerability (see Table 1).

**Table 1:** Dataset (THASV Index)

**Variables (Symbol)**	**Units**	**Levels**	**Points**	**Weighting Assignment**	**Datasets**	**Remarks**
Car ownership (CO)	Percentage of households that own two or more cars	Zonal—MSOAs	1–10	50%	Office for National Statistics (Calculated)	Higher car usage or petrol cost with high level of car ownership
Travel to work by car (TWC)	Percentage of car mode choice	Zonal—MSOAs	1–10		Office for National Statistics (Calculated)	Higher car usage or petrol cost with higher proportion of travel to work by car
Distance travelled to work by car (ADTW)	Travel time in minutes to the nearest employment centre by car	Zonal—MSOAs	1–10		Office for National Statistics (Calculated)	Higher car usage or petrol cost where journey distance to work is higher
Public transport accessibility levels (PTAL)	Public transport accessibility score	Zonal – LSOAs (Joint data provided by the authors from LSOAs to MSOAs)	1–10		Transport for London	Lower level of accessibility to a point on the public transport network will force residents to drive a car (higher car usage cost/petrol) due to lack of sufficient local public transport
Housing price vulnerability (HPV)	Median housing price	Zonal – MSOAs	1–10	50%	Land Registry	Housing cost affordability
Household income vulnerability (HIV)	Median household income	Zonal – MSOAs	1–10		Greater London Authority	Financial vulnerability relative to housing price
Index of multiple deprivation (IMD)	Index of multiple deprivation score	Zonal – LSOAs (Joint data provided by the authors from LSOAs to MSOAs)	1–10		Ministry of Housing, Communities and Local Government	The higher the scores, the worse the social inequity impacts
COVID-19 deaths (COVID)	Number of COVID-19 deaths per thousand	Zonal—MSOAs	N/A	N/A	Greater London Authority	COVID-19 deaths per 1,000 residents by MSOAs

*Note*: There were 983 Middle Layer Super Output Areas (MSOAs) in Greater London in 2011, which is the latest basic boundary map used in the analysis. Each MSOA has, on average, 4,000 households and 7,500 residents. LSOAs stands for Lower Layer Super Output Areas of which there are 4,835. Each LSOA contains an average of 700 households and 1,700 residents

1 $$I_{THASV}=W_{CO}X_{CO}+W_{TWC}X_{TWC}+W_{ADTW}X_{ADTW}+W_{PTAL}X_{PTAL}+W_{HPV/HIV}X_{HPV/HIV}+W_{IMD}X_{IMD},$$

where:

- *I*_*THASV*_: An aggregated composite index of travel, housing and social vulnerability.
- *X*_*CO*_: Percentage of households that own two or more cars.
- *X*_*TWC*_: Percentage of car mode choice.
- *X*_*ADTW*_: Travel time in minutes to the nearest employment centre by car.
- *X*_*PTAL*_: The reverse score of public transport accessibility.
- *X*_*HPV/HIV*_: Housing vulnerability score.
- *X*_*IMD*_: Index of multiple deprivation score.
- *W*_*i*_: Weighted index^2^.

**Table 2:** Descriptions of variables (MGWR)

**No**	**Variables**	**Description**	**Units**
1	TWC	Travel to work by car	Percentage
2	CO_2	Car ownership with two or more cars	Percentage
3	ADTW	Distance travelled to work by car	Number
4	PTAL	Public transport accessibility levels	Number
5	HPV	Housing price vulnerability	Number
6	HIV	Household income vulnerability	Number
7	IMD	Index of multiple deprivation	Number
8	HPR	Housing vulnerability ratio	Percentage
9	WORK	People who are working	Percentage
10	OLD	Older adults aged over 65	Percentage
11	HH	Households	Number
12	CHILD	Households with children	Percentage
13	NQ	No qualifications	Number
14	BHEALTH	Poor health	Percentage
15	RENT	Rent	Percentage
16	LONE	Lone parents not in employment	Percentage
17	PENSION	People aged over 60 who live in pension credit households	Percentage
18	ECO	Economically active	Number
19	DENSITY	Persons per hectare	Number
20	BAME	Black, Asian and minority ethnic	Percentage

The Bivariate Moran’s I was applied to assess the spatial autocorrelation and measure the influence that one variable *X* has on the occurrence of another variable *Z*, in close proximity, whereas the original Moran’s I statistic measured the degree of linear association between the values of a variable *X* in neighbouring districts (AURIN, 2021). The Bivariate Moran’s I statistic gives an indication of the degree of linear association between one variable *X*, and a different variable *Z*, in neighbouring districts *w*_*ij*_ *z*_*j*_ (but not in the same district). Therefore, the Bivariate Moran’s I can be computed as:

2 $$I^{BM}=\;\frac{N\sum_{i=1}^N\sum_{j=1}^Nw_{ij}x_iz_j}{S_0\sum_{i=1\;}^Nx_i^2},$$

where:

- *N* : The number of districts in the dataset.
- *S*_0_: The sum of the weights, which can be simplified to *A* if the spatial weight matrix is row-standardised.
- *x*_*i*_: The first variable, which is measured as the deviation from the mean.
- *z*_*i*_: The second variable, which is also measured as the deviation from the mean.
- *w*_*ij*_: The location variable for the area’s proximity, which is the element from the corresponding spatial weight matrix.

The multiscale geographically weighted regression (MGWR) was used to examine the potential key determinants associated with a high risk of COVID-19 infection/deaths from a socio-spatial analysis perspective. The relevant equation is shown below (Fotheringham et al, 2017):

3 $$Y_i=\;\sum_{j=0}^m\beta_{bwj}\left(g_i,\;h_i\right)X_{i,j}+\epsilon_i$$

Where:

- *Y*_*i*_: The number of COVID-19 deaths per thousand in the *i*^*th*^ area (i ∈{1,2,…,983} at location (*g*_*i*_ *, h*_*i*_)).
- *bwj:* The bandwidth applied for calibration of the *j* ^*th*^ conditional relationship.
- *β*_*j*_ *(g*_*i*_ *, h*_*i*_ *):* The *j*^*th*^ estimated regression parameters.
- *X*_*ij*_: The *j*^*th*^ explanatory variables (see Table 2).
- *ϵ*_*i*_: An error term.

**Fig. 1:**
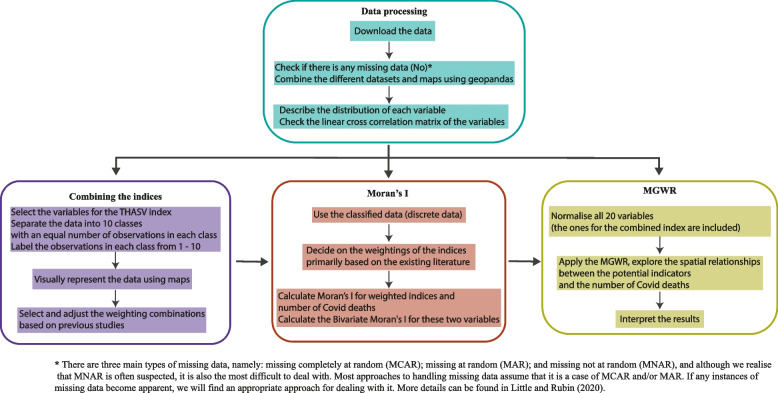
Workflow of the research methods

Figure 1 depicts a flow chart illustrating the research methods used in a step-by-step process.

### Data

Data used in this study were taken from the UK Census – Office for National Statistics, Transport for London (TfL), the Land Registry, the Ministry of Housing, Communities and Local Government (MHCLG), and Greater London Authority (see Table 1). The data provides information relating to travel, housing and social vulnerability, and the impacts of COVID-19. After cleaning and processing, the data was then used in conjunction with the basic boundary map at the MSOA level so that spatial and statistical analysis with visualisation could be carried out, using Greater London as a case study. All averaged individual data were calculated and aggregated by MSOAs. We also selected additional potential indicators (see Table 2) that were used in the multiscale geographically weighted regression (MGWR) in order to try to explain the geographical disparities in COVID-19 fatality rates. Data cleaning, processing, calculation and visualisation were carried out using Python and ArcGIS. In addition, the vulnerability indicators were chosen primarily based on the previous relevant studies carried out by Cao and Hickman (2018); Dodson and Sipe (2008); and Lovelace and Philips (2014), as well as being dependent on the dataset availability for the selected case study of Greater London.

## Findings and discussion

### Spatial descriptive statistics and visualisation

**Fig. 2:**
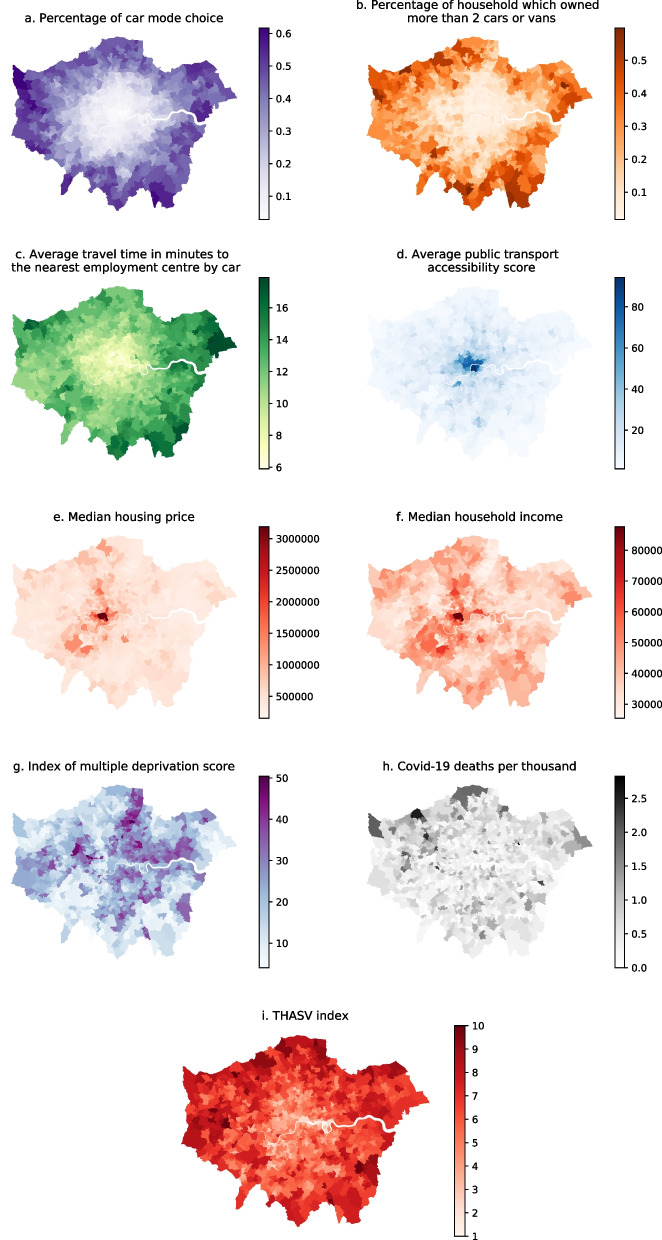
Choropleth map for each indicator and composite THASV index

In Fig. 2, we visually represented the data at MSOA levels in Greater London, which shows that, overall, there is a substantial knock-on effect between socio-spatial inequality, and a range of vulnerability issues, in particular when comparing districts in Inner London with those in Outer London. More specifically, Fig. 2a to d illustrate that there is a huge spatial difference in terms of travel vulnerability. The results show that residents living in suburban areas are more likely to experience issues relating to transport affordability with knock-on effects for social inequity, and socio-economic ‘oil vulnerability’ (Mattioli et al., 2018, 2019). This is primarily caused by the high levels of travel to work by car, car ownership, and long commuting times. Our findings are also in line with the existing research, which shows that travel and oil vulnerability mostly occur in suburban areas, such as in Sydney (Dodson & Sipe, 2007), Brisbane (Dodson et al., 2015), Hong Kong (Leung et al., 2018), and Yorkshire and the Humber (Lovelace & Philips, 2014).

Although it can be argued that London has the best public transport system (CityMonitor, 2016) and one of the largest urban transport networks in the UK, with integrated railways; the Underground; Docklands Light Railway; buses; road and river systems across the city, public transport provision and accessibility are disproportionately concentrated in central and Inner London, while Outer London and suburban areas are not as well served (e.g., see Fig. 2d). Therefore, based on the findings, we suggest that public transport networks should be developed in radial, orbital and tangential terms, particularly in the outer suburbs, in order to reduce people’s travel vulnerability. This would give people living in suburban areas a higher level of public transport service provision connecting to both central and surrounding areas and other suburban centres (Mees, 2010). In addition, active travel interventions (in Outer London), including a protected cycling infrastructure, should also be implemented to help reduce car ownership, and injuries sustained as a result of commuting (Adams & Aldred, 2020; Goodman et al., 2020), as well as helping to mitigate travel vulnerability.

With regards to housing affordability, Fig. 2e illustrates that even though some areas are shown in a light red colour, they remain unaffordable to the majority of residents, as the median housing price within these areas is valued at, at least £500,000. Housing prices in some parts of central and West London, such as Camden, Islington, Westminster and Kensington and Chelsea (upmarket districts), are even higher – more than £1,500,000 - which in turn leads to relatively higher rental rates, although the median household income is only around £38,000 per annum. Mortgages are usually available at between 3 and 4.5 times a household’s individual annual income. Furthermore, it can also be argued that some people may be forced to move to the outer suburbs or even beyond the boundaries of Greater London, with potential travel implications, such as ‘forced car ownership’ (Curl et al., 2018; Mattioli, 2017). However, housing vulnerability continues to be largely overlooked as a transport issue (Dewita et al., 2018; Mullen et al., 2020).

In Fig. 2.g, it can be seen that most socially deprived districts^3^ (especially those assessed in terms of poverty) are unevenly distributed across Greater London, and are particularly concentrated in East London. Additionally, Fig. 2h illustrates the number of COVID-19 deaths per thousand, and it shows that COVID-19 deaths are unevenly distributed throughout London, with relatively higher levels of deaths occurring in the Northwest of the city.

Regarding the neighbourhood ‘k’ value chosen, the supervised machine learning algorithm, k-nearest neighbours (kNN), has been widely applied to find the ‘k’ value of the appropriate nearest geographical neighbourhoods and measure these (Cover & Hart, 1967; García-Pedrajas et al., 2017; Wang et al., 2006). We, therefore, applied the same approach in this study.

### Spatial patterns and Moran’s I analysis

**Fig. 3:**
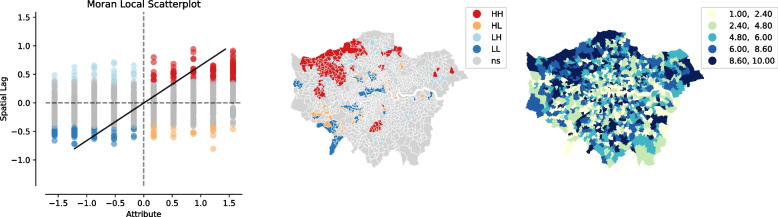
Moran’s I – COVID-19

According to Tobler’s First Law of Geography (1970: 236), “Everything is related to everything else, but near things are more related than distant things” (Tobler, 1970). With regard to COVID-19, Fig. 3 shows that not only are high levels of COVID-19 deaths distributed in Northwest London, but that this also has spatial effects on neighbouring districts. Therefore, based on these findings, further research could look into the relevant MSOAs at a disaggregated level in order to find out more about the reasons for this in greater detail.

**Fig. 4:**
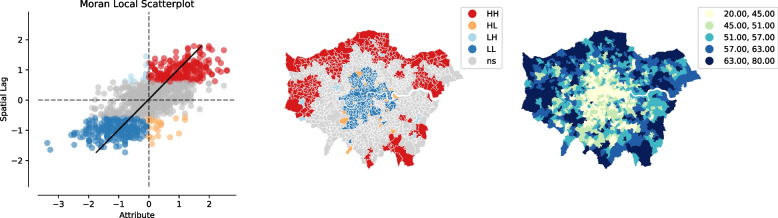
Moran’s I – THASV index

Regarding the THASV index, not surprisingly, we found that districts associated with high levels of travel, housing and social vulnerability are primarily distributed in the outer suburbs, and that this also has significant spatial impacts on neighbouring areas; whilst districts with low levels of vulnerability are mainly concentrated in Inner/Central London and this too has corresponding spatial effects on neighbouring districts (see Fig. 4).

**Fig. 5:**
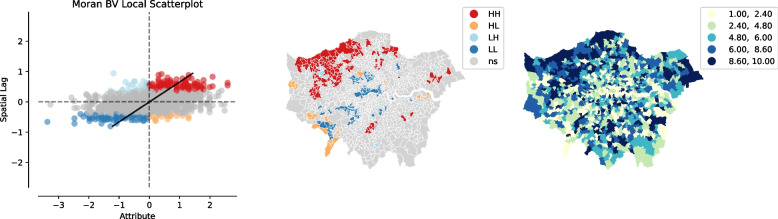
Bivariate Moran’s I – COVID-19 and THASV index

Finally, taking the aforementioned two variables into consideration simultaneously, our spatial analysis revealed that there are some positive correlations between COVID-19 deaths and the THASV index (Fig. 5). Furthermore, we also found that social deprivation is more likely to have potential impacts on issues relating to COVID-19.

### Multiscale geographically weighted regression

**Fig. 6:**
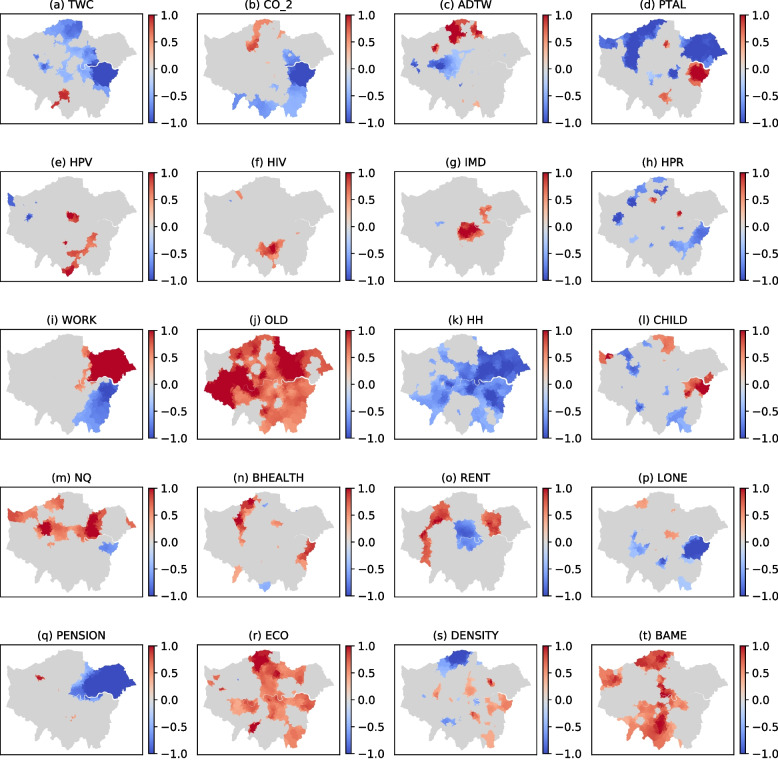
Results for the parameter estimates of the MGWR model

Finally, taking the aforementioned two variables into consideration simultaneously, our spatial analysis revealed some positive correlations between COVID-19 deaths and the THASV index (Fig. 6). This suggests that the impacts of COVID-19 exacerbate residents’ vulnerability, for example in the case of people living in Northwest London. Furthermore, we discovered that social deprivation is more likely to have an potential impact on issues relating to COVID-19.

Following the analyses described above, we then incorporated a spatial element into the analysis by using spatially weighted averages for each explanatory variable (Table 2), as well as taking neighbouring areas/effects into consideration using the MGWR model.

Overall, it can be seen from Fig. 6 that our results clearly capture the possibility that travel vulnerability and socio-economic indicators in a neighbouring district and the related vulnerability indices are strongly associated with the risk of infection and/or dying from COVID-19. The results also prove the significance of the spatial effects and transmission of COVID-19 in neighbouring areas (Guliyev, 2020; Pan et al., 2021). Furthermore, Fig. 6 shows that there is a substantial spatial inequality between each of the individual indicators and the risk of COVID-19 deaths across Greater London. In other words, the vulnerability in terms of both socio-economic differences and spatial inequalities should be taken into account when examining the potential effects of COVID-19 (Ehlert, 2021; Sun et al., 2021; Yang et al., 2021).

The summary statistics for the MGWR parameter estimates are displayed in Table 3. Overall, they show that the variables of travelling to work by car, being an older adult, the number of households in each MSOA, having no qualifications, being economically active, and coming from a Black, Asian or minority ethnic (BAME) background are more likely to have a significant association with COVID-19 infections/deaths.

**Table 3:** Summary statistics for MGWR parameter estimates (*n* = 983)

**Variables**	**Parameter Estimates**					
	**Mean**	**STD**	**Min**	**Median**	**Max**	**Adjusted t (95%)**
Intercept	0.12	0.19	-0.44	0.01	0.87	3.07
TWC	-0.35	0.40	-1.40	-0.39	0.93	3.09
CO_2	-0.06	0.44	-1.47	0.07	0.87	3.12
ADTW	-0.10	0.43	-1.52	-0.13	1.21	3.24
PTAL	-0.26	0.57	-1.92	-0.19	1.27	3.24
HPV	0.13	0.37	-1.02	0.10	1.54	3.16
HIV	0.07	0.30	-0.79	0.07	1.04	3.13
IMD	0.13	0.40	-0.64	0.09	1.30	3.15
HPR	-0.20	0.36	-1.83	-0.17	1.03	3.26
WORK	0.19	0.59	-1.08	0.13	2.46	3.19
OLD	0.84	0.33	0.22	0.74	2.57	3.23
HH	-0.60	0.28	-1.38	-0.59	0.16	3.28
CHILD	-0.02	0.39	-0.94	-0.05	1.31	3.23
NQ	0.32	0.45	-0.75	0.29	2.40	3.22
BHEALTH	0.17	0.30	-0.68	0.18	1.24	3.24
RENT	0.04	0.51	-0.94	0.04	1.29	3.14
LONE	-0.08	0.34	-1.27	-0.04	0.55	3.31
PENSION	-0.14	0.51	-1.56	0.04	0.97	3.15
ECO	0.46	0.31	-0.69	0.51	1.37	3.28
DENSITY	0.06	0.34	-1.45	0.10	1.00	3.27
BAME	0.39	0.33	-0.57	0.41	1.11	3.16
AIC	1936.86					
AICc	6943.03					
BIC	5626.12					
R^2^	0.91					

From a spatial perspective, car ownership and distance travelled to work by car in North London (Fig. 6b and c) and social deprivation in Inner London (Fig. 6g) all have a strong positive relationship with higher levels of COVID-19 infections/deaths. Not surprisingly, disadvantaged groups, such as older people aged over 65 (Fig. 6j), largely regardless of where they lived in London; people with no qualifications and poor health (Fig. 6m and n), particularly those located in North London, and BAME groups located in most parts of London are more likely to be at risk of dying from COVID-19 than their more affluent counterparts. Furthermore, higher levels of economically active zones are likely to result in a higher risk of COVID-19 deaths (Fig. 6r).

## Conclusions

In summary, this study has examined the combined issues of travel, housing and socio-economic vulnerability (THASV index), reflecting both social and spatial inequality, using Greater London as a case study with data analysed at the level of Middle Layer Super Output Areas (MSOAs).

Our findings show that most of the areas with high levels of composite vulnerability are located in Outer London, particularly in suburban areas. In addition, we also found a spatial correlation between the THASV index and COVID-19 deaths, which further exacerbates the potential implications of social deprivation and spatial inequality in Greater London. Furthermore, we found that social deprivation is more likely to significantly impact issues relating to COVID-19. These findings contribute to the existing literature on the impacts of COVID-19 and the resultant mobility and urban socio-spatial inequalities (Aoustin & Levinson, 2021; Cheng et al., 2021; De Vos, 2020; Jiao et al., 2021; Li et al., 2021; Rauws & van Lierop, 2020).

With regards to the theoretical and methodological contributions, our research primarily contributes to the existing literature by developing a composite socio-spatial regional vulnerability index (Cao, 2019; Cao & Hickman, 2018; Dodson & Sipe, 2007; Leung et al., 2018; Mattioli et al., 2019), that can be used to assess the combined issues of travel, housing and social vulnerability. Additionally, we tested the association between the key influential variables and geographical disparities in COVID-19 fatality rates.

In terms of policy implications, the findings can be used to inform sustainable city planning and urban development strategies designed to resolve urban socio-spatial inequalities and the related impacts of COVID-19, as well as guiding future policy evaluation of urban structural patterns. We also suggest that the new composite vulnerability index, ‘THASV’, can be replicated and applied to assess vulnerability-related issues in a wider context at a city level, in a range of other international cities. Furthermore, policymakers and urban planners should pay greater attention to the needs of individuals living in the high vulnerability regions identified on the map.

This research also has a few limitations. First, the indicators/variables included in the composite vulnerability index are quite limited due to the spatial level and data constraints. In addition, MSOAs were used to carry out the analysis in this research, and it might be helpful if a finer resolution geographical level, such as LSOAs, could be used in further research to examine spatial areas in greater detail (Cao & Hickman, 2018; Cuthill et al., 2019; Zhang et al., 2020). A nonlinear model could also be tested and applied (Xiao et al., 2021), together with advanced spatial methods to further explore urban patterns and dynamics (Gibbons et al., 2015; Ye & Liu, 2018, 2019), in future research.

## Data Availability

The data supporting the conclusions of this article can only be made available for academic research.

## Abbreviations

BAME: Black, Asian or minority ethnic
CDHA: Car dependence and housing affordability
GEV: Global eco-environmental vulnerability
kNN: K-nearest neighbours
MGWR: Multiscale geographically weighted regression
MHCLG: Ministry of Housing, Communities and Local Government
MSOA: Middle Layer Super Output Area
SDG: Sustainable development goal
TfL: Transport for London
THASV: Travel, housing and socio-economic vulnerability
VAMPIRE: Vulnerability assessment indices for mortgage, petrol and inflation risks and expenditure
VIPER: Vulnerability to rises in petrol expenses
VSD: Vulnerability scoping diagram

## Footnotes

1 The scope of this study primarily focuses on the development of a socio-spatial vulnerability index, rather than vulnerability relating to human activities, the natural environment and virtual embodiment.

2 The weightings of the indices were chosen primarily based on the rationale provided in the existing literature (see Cao and Hickman, 2018; Dodson and Sipe, 2008; Dodson et al., 2015; Leung et al., 2018) (see Table 2).

3 Indices of Multiple Deprivation (IMD) are widely applied to measure relatively deprived small areas in the UK. They consist of seven domains weighted as follows: income (22.5%); employment (22.5%); education (13.5%); health (13.5%); crime (9.3%); barriers to housing and services (9.3%); and living environment (9.3%) (Ministry of Housing, Communities and Local Government, 2019) (Noble et al., 2019).
